# PReS-FINAL-2014: Excellent results of the conventional treatment in a child with late-diagnosed juvenile dermatomyositis with severe calcinosis

**DOI:** 10.1186/1546-0096-11-S2-P27

**Published:** 2013-12-05

**Authors:** IP Nikishina, SV Arsenyeva, AN Shapovalenko

**Affiliations:** 1 Pediatric, Federal State Budgetary Institution "Research Institute of Rheumatology" under Russian Academy of Medical Science, Moscow, Russian Federation

## Introduction

Juvenile dermatomyositis (JDM) is a rare rheumatic disorder, often associated with poor functional outcome because of the high incidence of diffuse calcinosis in skeletal muscles, especially in case of late diagnosis.

## Objectives

To present the case report of the late-diagnosed JDM with severe calcinosis in a child with disease onset at 9 months.

## Methods

We have observed a female patient suffering from JDM since 9 months.

## Results

The disease started after revaccination against diphtheria and pertussis from symptoms of fever and muscle involvement (thickness and weakness) following a series of scheduled vaccinations (mumps, measles and rubella) clinical symptoms worsened, there were the typical skin changes, muscle weakness progressed, there were sporadic calcifications (8 months after disease onset), but the diagnosis was not established as a child experienced in primary care clinics. She was examined in our clinic after 3 years of disease onset when JDM was first diagnosed. The patient had fever, proximal muscle weakness, gait disturbance, typical cutaneous lesions (erythematous rash, discrete heliotrope of upper eyelids, Gottron's papules), slight enzymes elevation, nail fold changes by capillaroscopy and extensive diffuse calcinosis with significant muscle atrophy and lipodystrophy. The patient received oral prednisolone 1 mg/kg/day with slowly dose tapering and full cancellation after 4 years, methotrexate10 mg/m^2^/week till presence time, IVIG 1,5 g/kg monthly during the first 6 months and quarterly next two year. The treatment provided excellent results with rapid normalization of muscle strength, regression of skin and vascular lesions. The physical activity of the child and a relief of an inflammation led to the good development of muscle mass and displacement of calcifications to the surface of the skin with their localization in the larger conglomerates with its occasional spontaneous emptying.

## Conclusion

Unusual onset of JDM at a very early age, probably triggered by numerous vaccinations and lack of awareness about the disease general pediatricians contributes a significant delay in the diagnosis. But on the other hand there is an opportunity of good outcome of myopathy and calcinosis in infants with JDM

## Disclosure of interest

None declared.

